# The Microstructure and Mechanical Properties of Refractory High-Entropy Alloys with High Plasticity

**DOI:** 10.3390/ma11020208

**Published:** 2018-01-29

**Authors:** Yiwen Chen, Yunkai Li, Xingwang Cheng, Chao Wu, Bo Cheng, Ziqi Xu

**Affiliations:** 1 School of Materials Science and Engineering, Beijing Institute of Technology, Beijing 100081, China; yiwenchen_bit@sina.com (Y.C.); chengxw@bit.edu.cn (X.C.); wuchao19880724@hotmail.com (C.W.); hg2937772@163.com (B.C.); tapiki@126.com (Z.X.); 2 National Key Laboratory of Science and Technology on Materials under Shock and Impact, Beijing 100081, China

**Keywords:** RHEAs, alloys design, microstructure and mechanical properties

## Abstract

Refractory high-entropy alloys (RHEAs) are promising materials used at high temperature, but their low plasticity restricts their application. Based on the valence electron concentration (VEC) principle, four kinds of RHEAs (ZrTiHfV_0.5_Nb_0.5_, Zr_2.0_TiHfVNb_2.0_, ZrTiHfNb_0.5_Mo_0.5_, and ZrTiHfNb_0.5_Ta_0.5_) are designed (VEC < 4.5). The experimental results show that the plasticity of these alloys was greatly improved: the static compressive strain was higher than 50% at room temperature (RT), and some elongations were produced in the tensile process. Moreover, the microstructure and phase composition are discussed in detail. The addition of Nb, Mo, and Ta contributed to the high-temperature strength. Finally, the dynamic mechanical properties of these RHEAs with coordination between strength and plasticity are investigated.

## 1. Introduction

In recent years, a great number of new ideas have emerged in alloys [1]. High-entropy alloys (HEAs) were proposed by Yeh et al. [2,3], and have attracted much attention because of their extraordinary properties. HEAs are composed of five or more elements with equal or near-equal mole ratios (5–35 at %). In contrast with traditional theories, HEAs form a simple crystal structure for their high entropy. These alloys have good corrosion resistance [4,5], high hardness [6], and excellent thermal stability [7]. Besides, refractory high-entropy alloys (RHEAs) display good mechanical properties at a high temperature [8,9,10], as they contain refractory elements (Zr, Ti, Hf, V, W, Ta, Mo, et al.). For example, the MoNbTaVW alloy possesses a yield strength of 846 MPa, even at 800 °C [8]. However, it is difficult to machine most RHEAs at room temperature (RT), even though some RHEAs have reasonable compression strain [10,11,12].

The traditional workmanship for improving the deformability of RHEAs is usually adopted, such as changing the grain size and optimizing the distribution of the precipitation phase to modify the external microstructural appearance of alloys [13,14]. The intrinsic ductility of RHEAs also requires honing in on the alloy’s design process. For stress, dislocation nucleation requires that the local shear stress reaches the ideal values for a perfect crystal. Nevertheless, the initial crack occurs when the local tensile stress perpendicular to the dissociation plane exceeds the ideal tensile strength in metals. This phenomenon causes the activation of dislocation nucleation before the crack formation, which is called shear instability [15]. In this case, the alloys have some inherent plasticity, in that some slip was produced by dislocation. The metals in groups 4 and 5 of the periodic table have a good plasticity due to their shear instability process. For the metals in group 6, the tensile fracture process finishes before the shear instability process starts, leading to their low plasticity.

From the perspective of bonding energy, the shear instability process easily occurs when the total energy of alloys is relatively lower. According to the Jahn–Teller deformation [16,17], the bonding energy of alloys decreases through the splitting of the near degenerate orbital, and decreasing the valence electron concentration (VEC) can increase the driving force of the Jahn–Teller deformation. Consequently, alloys with a low VEC are conductive to the occurrence of shear instability. Previous studies have illustrated that RHEAs possess good plasticity when the VEC is lower than 4.5 [17].

In this paper, four kinds of RHEAs are designed according to the above VEC principle. The static compressive strain of these alloys was higher than 50% and unfaulted at RT. Some elongations were produced in the tensile process, and the tensile strain decreased with increasing VEC. In addition, high-temperature and dynamic mechanical properties are discussed.

## 2. Experimental Methods

The ingots of four kinds of RHEAs were prepared by arc melting under a purified argon atmosphere. The cooling system employed a water-cooled copper crucible. Due to the high melting point of these alloys, relatively large current (350–450 A) and voltage (12–15 V) were used in the melting process. The low-melting metals were placed lower at the bottom and the ingots were remelted at least six times, which guaranteed the homogeneity of the composition. The shape of these ingots was similar to a button, and the height of the thickest part was about 14 mm. The crystal structure was characterized with an X-ray diffractometer with Cu$-K_{\alpha }$ radiation. The 2-theta scan ranged from 20 degrees to 90 degrees at a speed of 0.2 s/step. After being polished and etched (the corrosive liquid consisted of HF, HNO_3_, and H_2_O), the specimens were observed in the metallographic microscope. A scanning electron microscope (SEM, HITACHIS4800, Tokyo, Japan,) was used to analyze the microstructure.

The microhardness of RHEAs was measured by a 450SVD^TM^ Vicker’s hardness tester (London, UK) under the load of 300 g (applied for 15 s). The ultimate hardness was averaged by five values, which were tested in different positions. The static compressive properties were tested on the Instron 5569 electronic universal testing machine (Boston, MA, USA) with a strain rate of 10^−3^ S^−1^. The specimen was the shape of a cylinder (φ 4 mm × 6 mm). The tensile properties were tested on a tensile testing machine produced by Instron Company (Boston, MA, USA). The size of the tensile sample adopted was GB/T228-2002. The high-temperature compressive performances were performed on a Gleeble-3500 thermal simulator (Poestenkill, New York, NY, USA) with a Pt-Rh alloys thermocouple. The specimen was the shape of a cylinder (φ 6 mm × 9 mm) and the heating rate was set at 300 °C/min. Each alloy was heated to 700 °C, 800 °C, and 900 °C in turns, and the holding time was three minutes. The dynamic compressive properties were tested on the split Hopkinson pressure bar (SHPB). The specimens were the shape of a cylinder (φ 4 mm × 4 mm), and each alloy was loaded under 0.3 atmospheres, 0.4 atmospheres, and 0.5 atmospheres. In order to ensure the accuracy of the experimental data, two ends of the specimens were rubbed down to make them parallel to each other in the course of the experiments.

## 3. Results and Discussion

### 3.1. Crystal Structure and Microstructure Analysis

**Figure 1:**
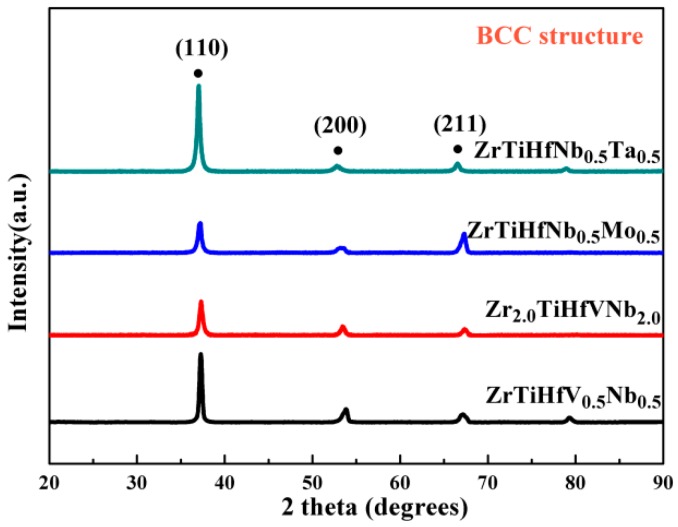
X-ray diffraction patterns of the refractory high-entropy alloys (RHEAs).

The typical X-ray diffraction patterns of these RHEAs are shown in Figure 1. The peaks are consistent with the single phase of the body-centered cubic (BCC) structure without any other phases. In the alloys’ solidification process, the phase formation is related to Gibbs free energies of different phases in equilibrium. Because of the high configuration entropy in some RHEAs, the solid solution phase with the lowest energy can stay stable at RT [18,19]. Accordingly, the phase can be predicted in the alloys’ design process. The atomic-size difference (δ) and the single-phase formation ability (Ω) combining the effects of ΔS_mix_ and ΔH_mix_ [20] are calculated and displayed in Table 1; they are in the range of the single-phase formation.

**Figure 2:**
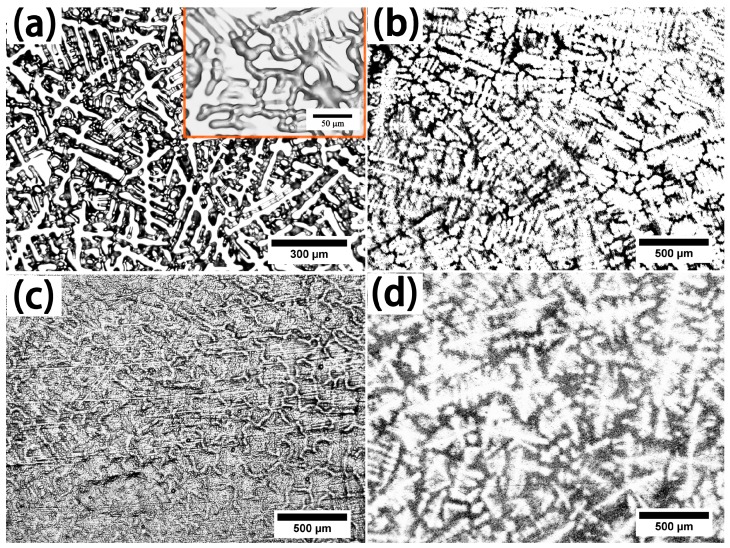
The microstructures of the as-cast RHEAs after being etched: (**a**) ZrTiHfV_0.5_Nb_0.5_; (**b**) Zr_2.0_TiHfVNb_2.0_; (**c**) ZrTiHfNb_0.5_Mo_0.5_; (**d**) ZrTiHfNb_0.5_Ta_0.5_.

The metallography of these as-cast RHEAs is shown in Figure 2. Four kinds of RHEAs display dendritic structures, but each composition has a different morphology. In Figure 2a, the primary dendrites intersect with secondary dendrites, and the dendrites are distributed as a network in the high-magnification images. As shown in Figure 2b, the secondary dendrites grow from both sides of the primary dendrites, and some dendrites have a large size in the after-solidified region. The microstructure of the ZrTiHfNb_0.5_Mo_0.5_ alloy displays reticulate dendrites in Figure 2c, which is beneficial to plasticity and strength. Figure 2d demonstrates the relatively large dendrite structure of the ZrTiHfNb_0.5_Ta_0.5_ alloy. The dendrites are not connected, and the secondary dendrites are perpendicular to the primary dendrite, leading to the low strength of these alloys.

**Figure 3:**
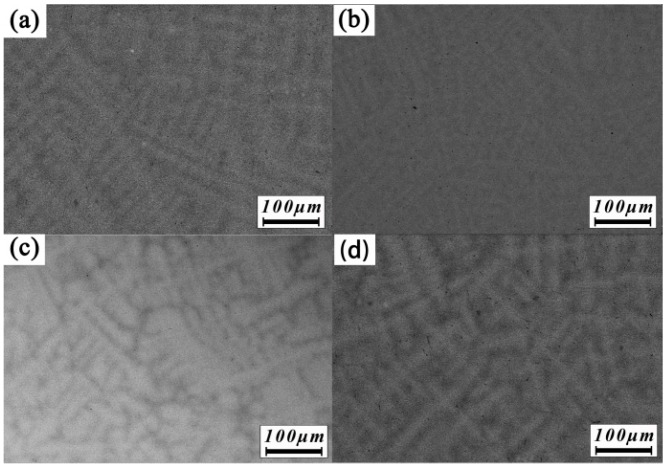
Backscattered electron (BSE) images of the as-cast RHEAs: (**a**) ZrTiHfV_0.5_Nb_0.5_; (**b**) Zr_2.0_TiHfVNb_2.0_; (**c**) ZrTiHfNb_0.5_Mo_0.5_; (**d**) ZrTiHfNb_0.5_Ta_0.5_.

The components results of these alloys for different regions are displayed in Table 2, and the backscattered electron images are shown in Figure 3. The degree of segregation in the alloys is characterized by the segregation ratio (*S_R_*):

(1) $$S_{R}=C_{B}^{i}/C_{A}^{i}$$

where $C_{A}^{i}$ is the atomic percentage of the *i*th element in dendrites, and $C_{B}^{i}$ is in the interdendrites. In the ZrTiHfV_0.5_Nb_0.5_ and Zr_2.0_TiHfVNb_2.0_ alloys, the five elements with a similar melting point are distributed evenly in the dendrites and interdendrites. With the addition of Mo and Ta with high melting, there is a clear segregation in the ZrTiHfNb_0.5_Mo_0.5_ and ZrTiHfNb_0.5_Ta_0.5_ alloys. Zr, with a low melting point, is gathered in the interdendrites, and Mo and Ta are concentrated in the dendrites. The melting point of Ti and Hf is moderate, and these are distributed uniformly in the dendrites and interdendrites.

### 3.2. Static Compression Properties and Hardness Analysis

**Figure 4:**
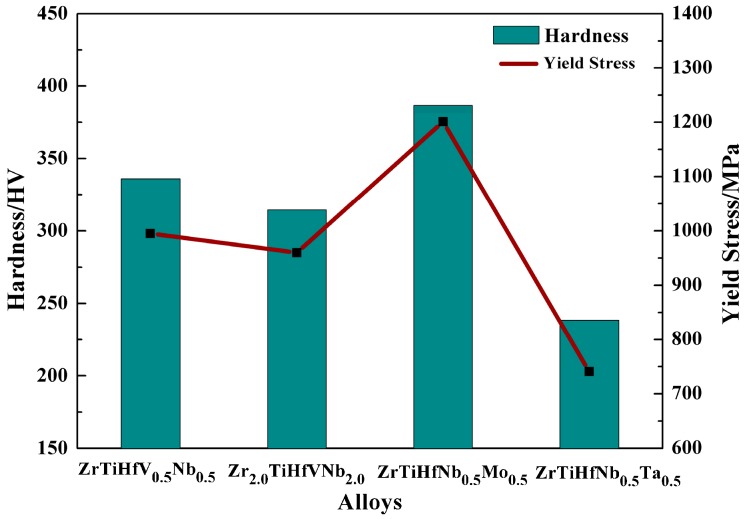
The hardness and compression yield stress of these RHEAs.

**Figure 5:**
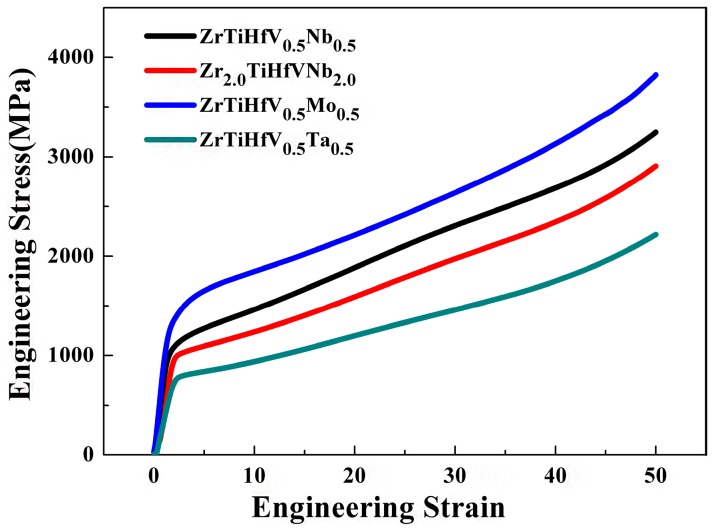
The static compression engineering curves of the RHEAs.

The yield stress of the static compression and hardness are displayed in Figure 4, and the stress–strain curves at RT are shown in Figure 5. These alloys were unfaulted in the processes of experiments (>50%). The deformation underwent the elastic deformation and plastic deformation stages. A certain degree of hardening occurred as the strain increased. The yield stress of the ZrTiHfNb_0.5_Mo_0.5_ alloy was the highest insofar as the bond energy between Mo and other elements (Zr, Ti, Hf, V, and Nb) is relatively bigger. In comparison with the ZrTiHfNbMo alloy, whose strain was only 9% [21], the ZrTiHfNb_0.5_Mo_0.5_ alloy, whose strain is over 50%, is more convenient for machines due to its high plasticity. The yield stress of the ZrTiHfV_0.5_Nb_0.5_ alloy was 995 MPa, which is an improvement of nearly 300 MPa when compared with the ZrTiHfVNb alloy [22].

**Figure 6:**
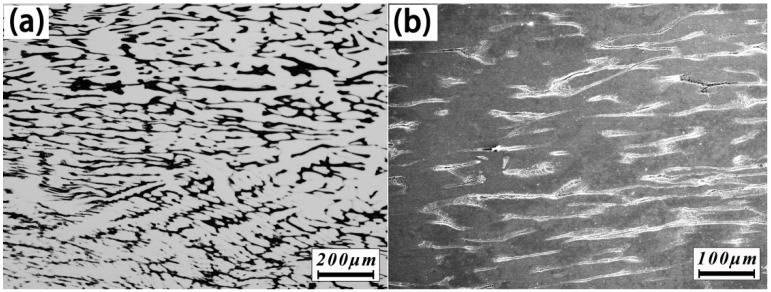
The metallographs of the ZrTiHfV_0.5_Nb_0.5_ alloys after being compressed: (**a**) Optical microscope (OM); (**b**) Scanning electron microscope (SEM).

The section images of the ZrTiHfV_0.5_Nb_0.5_ alloy after the compression are shown in Figure 6. The as-cast dendrites display a streamline structure, and the secondary dendrites tend to be paralleled to primary dendrites. Although a small quantity of cracks are observed in Figure 6b, many dendrites remain complete comparing Figure 6a with Figure 2a, which indicates that the deformability of the ZrTiHfV_0.5_Nb_0.5_ alloy was perfect.

### 3.3. Tensile Properties Analysis

**Figure 7:**
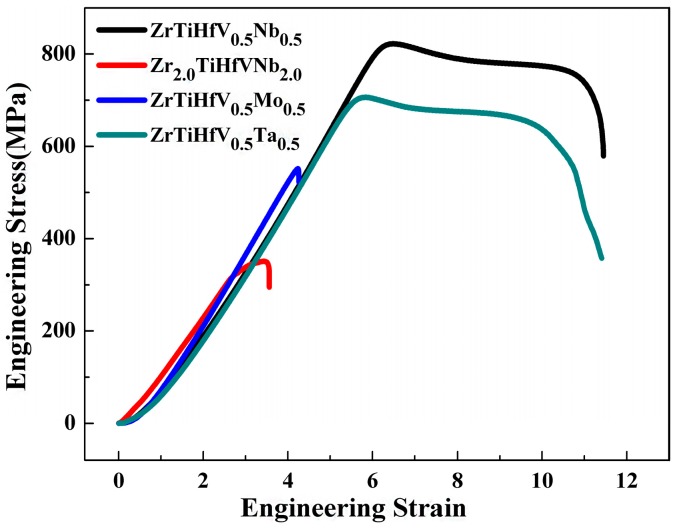
The tension stress–strain curves of the RHEAs.

**Figure 8:**
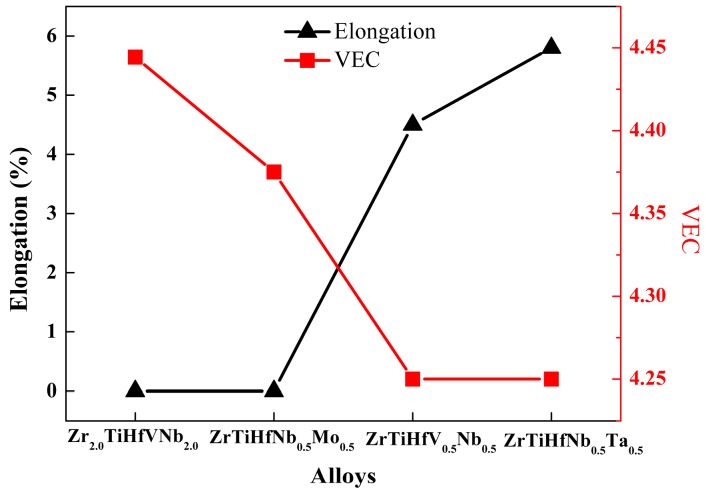
The relationship between the valence electron concentration (VEC) and tensile elongation of different RHEAs.

The stress–strain curves of the tensile experiments are shown in Figure 7. The ZrTiHfV_0.5_Nb_0.5_ and ZrTiHfNb_0.5_Ta_0.5_ alloys underwent a similar process of elastic and plastic deformation, and finally fracture. The tensile stress and elongations of the ZrTiHfV_0.5_Nb_0.5_ and ZrTiHfNb_0.5_Ta_0.5_ alloys were 820 MPa and 4.5%, and 696 MPa and 8%, respectively. The Zr_2.0_TiHfVNb_2.0_ and ZrTiHfNb_0.5_Mo_0.5_ alloys failed in the elastic region by a stress level of 349 MPa and 530 MPa, respectively. The VEC of the RHEAs is calculated as:

(2) $$\mathrm{VEC}=\sum C_{i}*{\mathrm{VEC}}_{i},$$

where $C_{i}$ is the atomic percentage of the *i*th element and $VEC_{i}$ is its valence electron. Figure 8 shows the relationship between the VEC and the tensile strain of the researched RHEAs. It shows that the VEC and tensile strain have an opposite trend, which reveals that the RHEAs with good plasticity can be obtained by controlling the VEC.

### 3.4. High-Temperature Compressive Properties

**Figure 9:**
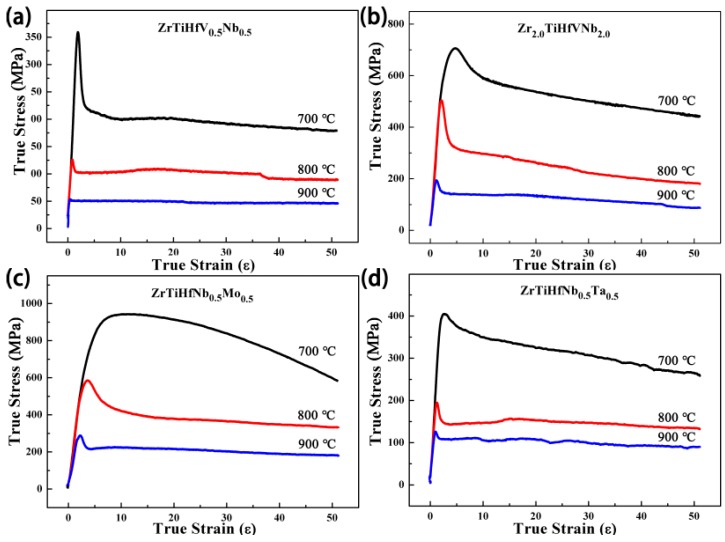
The high-temperature compression stress–strain curves of the alloys: (**a**) ZrTiHfV_0.5_Nb_0.5_; (**b**) Zr_2.0_TiHfVNb_2.0_; (**c**) ZrTiHfNb_0.5_Mo_0.5_; (**d**) ZrTiHfNb_0.5_Ta_0.5_.

The compressive curves of the ZrTiHfV_0.5_Nb_0.5_, Zr_2.0_TiHfVNb_2.0_, ZrTiHfNb_0.5_Mo_0.5_, and ZrTiHfNb_0.5_Ta_0.5_ alloys tested at high temperature (700 °C, 800 °C, and 900 °C) are shown in Figure 9. The stress increased rapidly and then decreased to a stable value with an increase in deformation. The stress of the alloys was closely related to the temperature. As a high temperature could provide much more energy, it would promote a movement of dislocation, vacancy, and grain boundary, which could in turn decrease the stress. The high-temperature yield stress of the ZrTiHfV_0.5_Nb_0.5_ alloy was 337 MPa at 700 °C, 116 MPa at 800 °C, and 46 MPa at 900 °C. When adjusting the content of Nb, the yield stress of the Zr_2.0_TiHfVNb_2.0_ alloy was 647 MPa at 700 °C, 476 MPa at 800 °C, and 165 MPa at 900 °C. Because Mo has good ability of softening resistance at high temperature, the yield stress of the ZrTiHfNb_0.5_Mo_0.5_ alloy remained at 937 MPa at 700 °C, 539 MPa at 800 °C, and 274 MPa at 900 °C. It is therefore a promising candidate in the high-temperature alloy category. Although Ta has the highest melting point, the yield stress of the ZrTiHfNb_0.5_Ta_0.5_ alloy was 383 MPa at 700 °C, 170 MPa at 800 °C, and 112 MPa at 900 °C due to the low strength of Ta.

**Figure 10:**
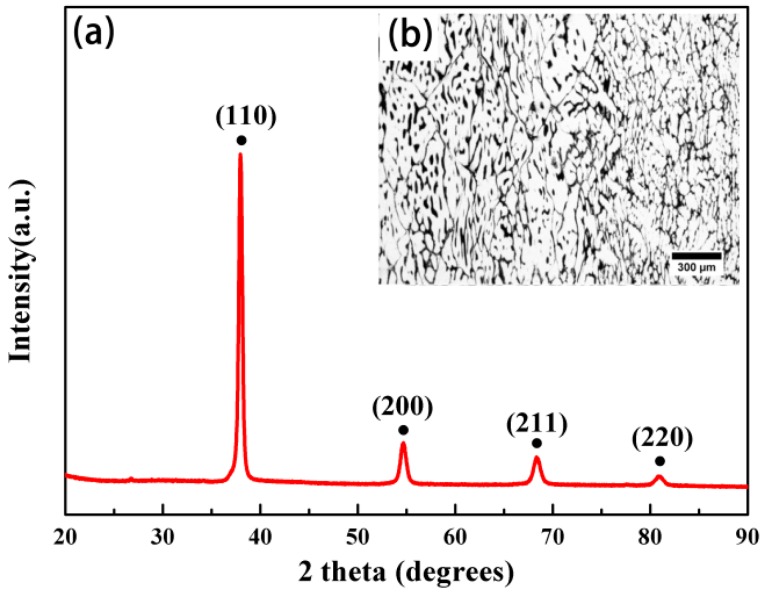
The microstructures of the ZrTiHfNb_0.5_Ta_0.5_ alloys after being compressed at 800 °C: (**a**) XRD pattern; (**b**) metallography.

In Figure 10a, no other phase was produced during the heating process (800 °C) in the ZrTiHfNb_0.5_Ta_0.5_ alloy. Figure 10b shows the post-compression metallograph. The original grain was compressed, and a small amount of equiaxial grains gathered along the direction of the deformation, which is a typical fracture after a high-temperature compression.

The density and compressive properties of the prepared RHEAs and other RHEAs from previous research are shown in Table 3. The strength matches well with plasticity by controlling the VEC. With regard to high-temperature stress, the special strength of the ZrTiHfNb_0.5_Mo_0.5_ alloy at 800 °C was relatively higher, and the application has apparent preponderance in the elevated field.

### 3.5. Dynamic Compressive Properties Analysis

**Figure 11:**
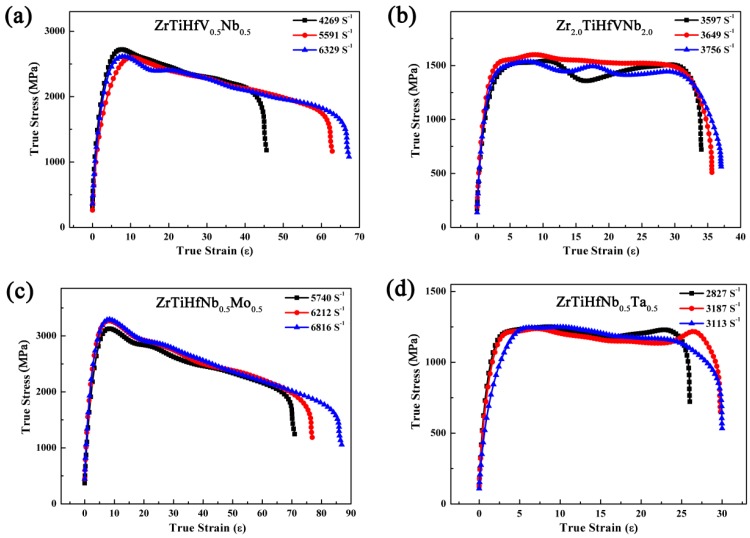
The dynamic compression stress–strain curves of the RHEAs: (**a**) ZrTiHfV_0.5_Nb_0.5_; (**b**) Zr_2.0_TiHfVNb_2.0_; (**c**) ZrTiHfNb_0.5_Mo_0.5_; (**d**) ZrTiHfNb_0.5_Ta_0.5_.

The dynamic compressive curves of RHEAs are exhibited in Figure 11. Each alloy is loaded by 0.3 atmospheres, 0.4 atmospheres, and 0.5 atmospheres. At the same pressure, the strain rate of these alloys is obviously different, but the yield stress of these alloys has no obvious change with the strain rate increase. The stress displays a rapid uptrend because the strain hardening and strain rate strengthening are dominant in the plastic stage. With the rapid deformation increase, the heat could not dissipate, which would have led to the materials’ thermal softening. In this condition, the stress of the alloys is the result of both heat softening and the reinforcement produced by strain. In the ZrTiHfV_0.5_Nb_0.5_ and ZrTiHfNb_0.5_Mo_0.5_ alloys, the stress decreases slightly with the strain increase because the heat softening effect is preponderant. However, the strengthening and softening effects remain balanced in the Zr_2.0_TiHfVNb_2.0_ and ZrTiHfNb_0.5_Ta_0.5_ alloys, leading the stress to remain the same.

In Table 4, the yield stress is higher than the static condition, but the energy absorption of the four RHEAs is apparently different. In the dynamic deformation process, these alloys with coordination between strength and plasticity undergo a large deformation and energy absorption. The average stress of the ZrTiHfNb_0.5_Mo_0.5_ alloy is 3337 MPa, and the energy absorption is 8.46 MJ·m^−2^, thereby providing a promising material under dynamic conditions. Although the plasticity of the ZrTiHfNb_0.5_Ta_0.5_ alloy is high in the static condition, the strain is only 24.86% in the dynamic deformation process due to its low strength.

**Figure 12:**
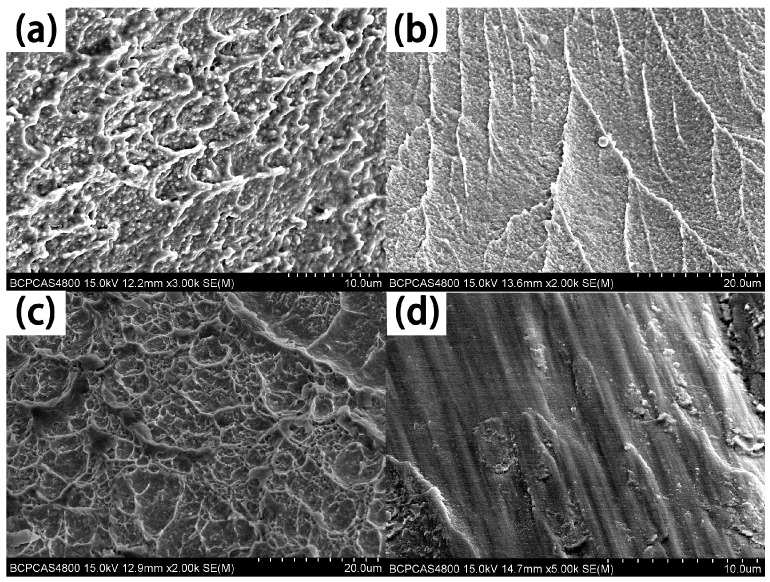
Scanning electron microscopy (SEM) images of these RHEAs after being dynamically compressed and etched: (**a**) ZrTiHfV_0.5_Nb_0.5_; (**b**) Zr_2.0_TiHfVNb_2.0_; (**c**) ZrTiHfNb_0.5_Mo_0.5_; (**d**) ZrTiHfNb_0.5_Ta_0.5_.

The fracture surface is shown in Figure 12. The fracture morphology of the ZrTiHfV_0.5_Nb_0.5_ alloy had some shallow tough fossae, and part of the section was comparatively smooth (Figure 12a), which indicates that the fracture model of the alloys was a tough-brittle fracture. With the Zr and Nb increase, the fracture surface of the Zr_2.0_TiHfVNb_2.0_ alloy exhibited river-like features (Figure 12b). When adding Mo in alloys, the fracture appearance uniformly distributed the tough fossae (Figure 12c). The fracture surface of ZrTiHfNb_0.5_Ta_0.5_ alloy was smooth (Figure 12d), which shows that its plasticity was poor under dynamic conditions.

## 4. Conclusions

In this paper, four kinds of RHEAs with high plasticity (ZrTiHfV_0.5_Nb_0.5_, Zr_2.0_TiHfVNb_2.0_, ZrTiHfNb_0.5_Mo_0.5_, and ZrTiHfNb_0.5_Ta_0.5_) were designed by controlling the VEC, and they were synthesized via vacuum arc melting in order to certify the accuracy of the VEC principle. Based on the results we obtained and the subsequent analysis, we drew the following conclusions:

(1) The crystal structure of the RHEAs was identified as a BCC structure and the dendrites were found in the optical microscope.
(2) The compressive strain of these alloys was higher than 50% and the compressive strength was relatively higher. The RHEAs with the minimum VEC exhibited the maximum tensile deformation.
(3) The addition of Nb, Mo, and Ta contributed to a high-temperature strength.
(4) It would be beneficial to explore new-type RHEAs with good plasticity by controlling the VEC. RHEAs that combine good plasticity and high strength would be convenient for processing and manufacturing.

## Figures and Tables

**Table 1:** The relevant thermodynamic parameters and valence electron concentration (VEC) of the refractory alloys.

Alloy	Ω	Delta/%	H_mix_ ($KJ\cdot mol^{-1}$)	S_mix_	T_m_ (K)
ZrTiHfV_0.5_Nb_0.5_	2.58	5.88	−9.44	12.9671	2257
Zr_2.0_TiHfVNb_2.0_	2.19	6.05	−11.77	12.8860	2336
ZrTiHfNb_0.5_Mo_0.5_	1.62	5.10	−16.70	12.9671	2349
ZrTiHfNb_0.5_Ta_0.5_	2.83	4.26	−9.44	12.9671	2398

**Table 2:** The components analysis of the RHEAs.

Alloys	Region	Zr	Ti	Hf	V	Nb	Mo	Ta
ZrTiHfV_0.5_Nb_0.5_	Dendrite	24.31	24.34	25.91	12.03	13.42	-	-
	Interdendrite	22.35	23.74	26.7	13.32	13.89	-	-
	*S_R_*	0.92	0.98	1.03	1.11	1.04	-	-
Zr_2.0_TiHfVNb_2.0_	Dendrite	27.57	13.58	14.65	13.4	30.8	-	-
	Interdendrite	25.92	13.72	14.8	14.61	30.97	-	-
	*S_R_*	0.94	1.01	1.01	1.09	1.01	-	-
ZrTiHfNb_0.5_Mo_0.5_	Dendrite	22.99	21.85	25.58	-	18.99	10.59	-
	Interdendrite	25.95	20.37	25.6	-	20.35	7.73	-
	*S_R_*	1.13	0.93	1	-	1.07	0.73	-
ZrTiHfNb_0.5_Ta_0.5_	Dendrite	23.56	21.36	23.33	-	17.5	-	10.78
	Interdendrite	28.53	21.47	23.69	-	16.38	-	6.96
	*S_R_*	1.21	1.01	1.02	-	0.94	-	0.65

**Table 3:** The density and mechanical properties at room temperature (RT) and high temperature compared with referenced literature.

Alloys	Density/g·cm^−3^	Strain/%	Yield Stress/MPa	Specific Strength/MPa·cm^3^/g	Elevated Temperature Strength (800 °C)	Specific Strength/MPa·cm^3^/g (800 °C)	VEC
CrNbTiZr	6.70	6%	1260	188.06	300	44.78 [23]	4.75
HfMoNbTiZr	8.69	9%	1575	181.24	635	73.07 [21]	4.60
HfNbTiVZr	8.06	30%	1170	145.16	408	50.62 [22]	4.40
HfNbTaTiZr	9.94	>50%	929	93.46	535	53.82 [9]	4.40
ZrTiHfV_0.5_Nb_0.5_	8.08	>50%	990	122.51	125	15.47	4.25
Zr_2.0_TiHfVNb_2.0_	7.83	>50%	956	122.17	507	64.79	4.43
ZrTiHfNb_0.5_Mo_0.5_	8.43	>50%	1195	141.77	595	70.59	4.38
ZrTiHfNb_0.5_Ta_0.5_	9.14	>50%	738	80.79	193	21.13	4.25

**Table 4:** The dynamic compressive mechanical properties and energy absorption of the RHEAs.

Alloys	Average Stress/MPa	Average Strain/%	Energy Absorption/(MJ·m^−2^)		
			0.3 Atm	0.4 Atm	0.5 Atm
ZrTiHfV_0.5_Nb_0.5_	2642	50.03	4.21	4.08	5.71
Zr_2.0_TiHfVNb_2.0_	1594	31.98	1.93	2.09	2.04
ZrTiHfNb_0.5_Mo_0.5_	3337	70.1	7.02	7.78	8.46
ZrTiHfNb_0.5_Ta_0.5_	1237	24.86	1.22	1.36	1.34
